# Anti-metabolic glutamate receptor 5 encephalitis with gangliocytoma: a case and review of the literature

**DOI:** 10.1186/s12883-024-03528-z

**Published:** 2024-01-13

**Authors:** Kaili Shi, Huimin Zhao, Ying Li, Xiaojing Li, Wenxiong Chen

**Affiliations:** 1 Department of Neurology, Guangzhou Women and Children’s Medical Center, Guangzhou, 510623 China; 2 Department of Pediatrics, People’s Hospital of Pidu, Sichuan, 611730 China

**Keywords:** Autoimmune encephalitis, Glutamate receptor, Gangliocytoma, Paraneoplastic syndrome, Children

## Abstract

**Background:**

There are very limited reports on anti-metabolic glutamate receptor5 (mGluR5) encephalitis, especially lacking of pediatric research. The disease was mostly accompanied by tumors, mainly Hodgkin's lymphoma. No reports of other tumors, such as gangliocytoma have been reported to associate with anti-mGluR5 encephalitis so far.

**Case presentation and literature reviews:**

We reported a case of a 12-year-old boy with anti-mGluR5 encephalitis complicated with gangliocytoma. The patient suffered from mental disorders including auditory hallucination, and sleep disorders. His cranial magnetic resonance imaging (MRI) showed an abnormality in the right insular lobe. Autoimmune encephalitis antibodies testing was positive for mGluR5 IgG antibody both in cerebrospinal fluid and serum (1:3.2, 1:100 respectively). Abdominal CT indicated a mass in left retroperitoneal confirmed with gangliocytoma via pathology. The patient underwent resection of gangliocytoma. After first-line immunotherapy (glucocorticoid, gamma globulin), his condition was improved. Furthermore, we provide a summary of 6 pediatric cases of Anti-mGluR5 encephalitis. Most of them complicated with Hodgkin's lymphoma, except the case currently reported comorbid with gangliocytoma. The curative effect is satisfactory.

**Conclusions:**

We report the first patient with anti-mGlur5 encephalitis complicated with gangliocytoma. It suggests that in addition to paying attention to the common lymphoma associated with anti-mGlur5 encephalitis, we should also screen the possibility of other tumors for early detection of the cause, active treatment and prevention of recurrence.

## Background

The term “Ophelia syndrome” was first used by Carr in 1982 to describe the limbic encephalitis in his daughter associated with Hodgkin’s lymphoma [1]. It was until 2011 that the metabolic glutamate receptor5 (mGluR5) antibody was finally identified in this syndrome in two patients [2]. The disease was mostly accompanied by tumors, mainly Hodgkin's lymphoma. There are limited numbers of mGluR5 encephalitis, cases in children are particularly rare. No reports of other tumors, such as gangliocytoma have been reported to associate with anti-mGlur5 encephalitis so far. This paper reported a case of gangliocytoma-associated autoimmune encephalitis (AE) with positive mGluR5 antibodies in a boy, which suggests clinicians to pay attention to the possibility of anti-mGluR5 encephalitis associated with other tumors except Hodgkin's lymphoma.

## Case presentation

The patient was a 12-year-old boy. He was admitted to hospital on May 06, 2022. The patient had intermittent headache 20 days ago, with intermittent fever 10 days ago, accompanied by mental symptoms such as irritability, auditory hallucinations, and sleep disorders. His memory and comprehension decreased. He had auditory hallucination. The muscle force, muscle tone of limbs and tendon reflexes were normal. The coordination movement and sensation detection were normal. Meningeal stimulation signs and pathological signs were negative.

**Fig. 1:**
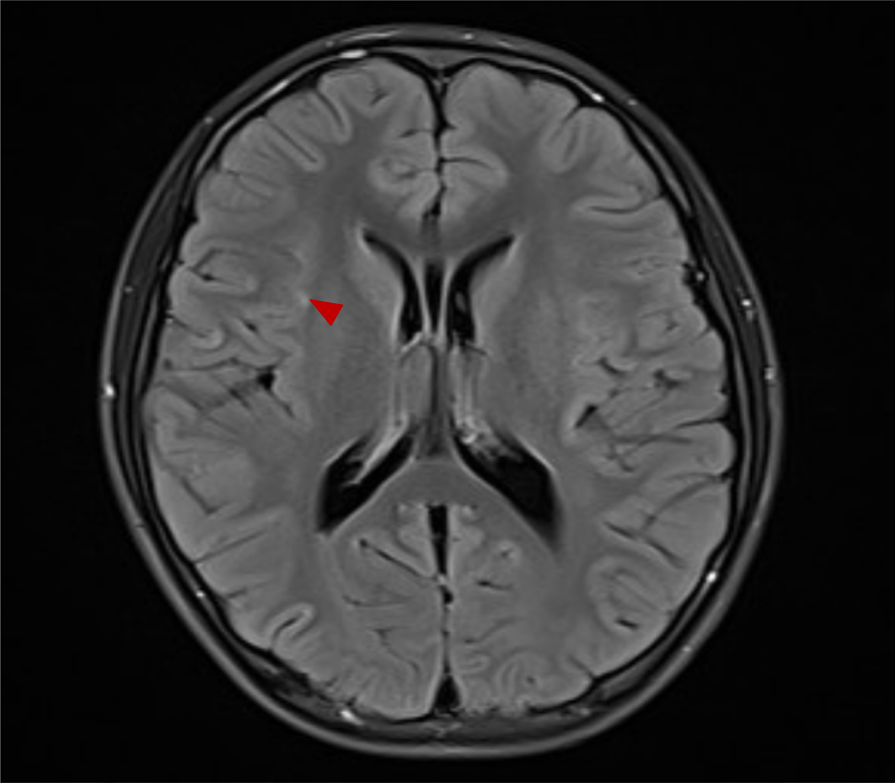
The brain MRI image of the patient. Red arrow showed a few speckled abnormal enhanced signal shadows in the right insular lobe

**Fig. 2:**
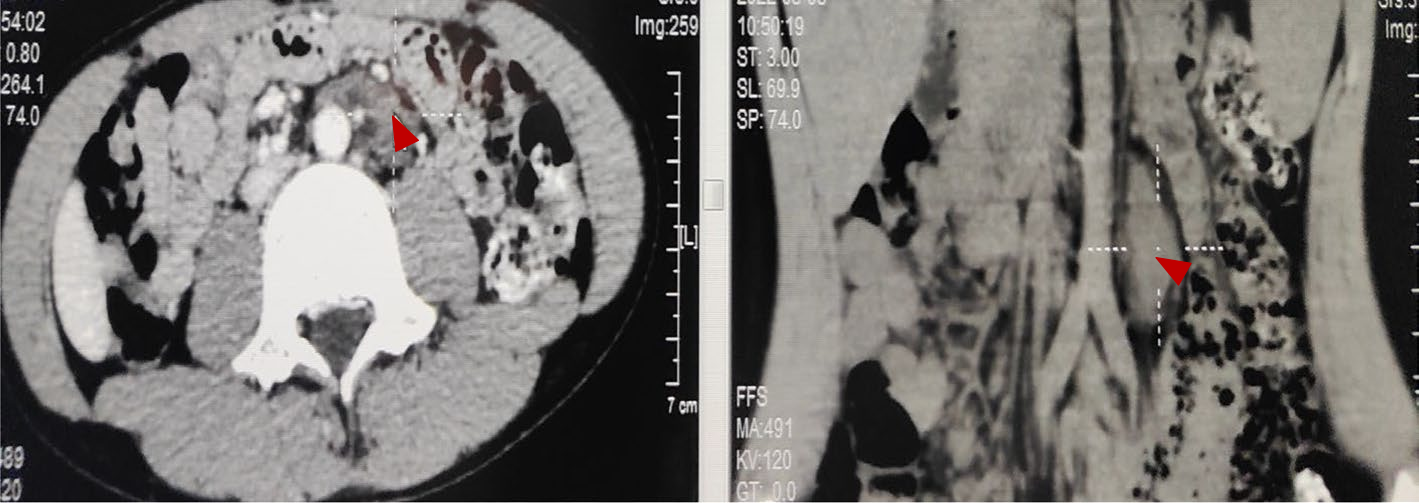
Abdominal CT images of the patient. Red arrows showed a soft tissue mass on the left side of the retroperitoneal abdominal aorta (3.8*2.0*1.8 cm)

**Fig. 3:**
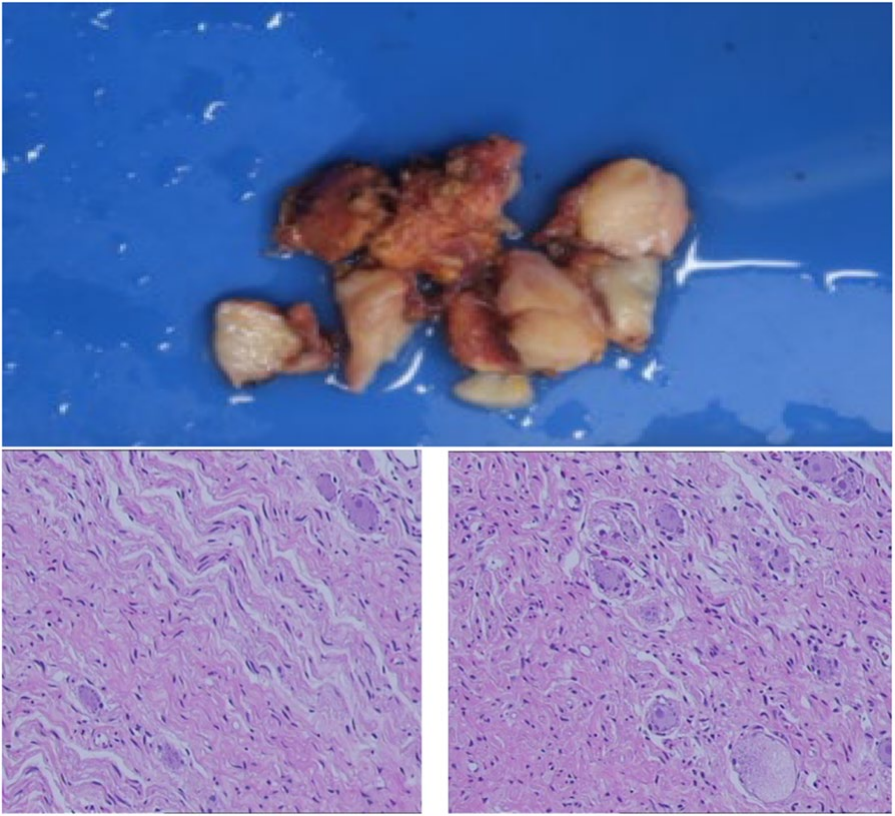
Pathological examination of the abdominal mass suggested gangliocytoma, mature and medium-sized

**Fig. 4:**
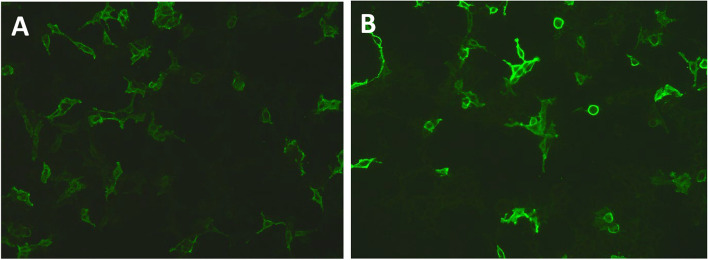
Testing of cerebrospinal fluid and serum for antibodies to metabotropic Glutamate receptor 5 was positive on cell based assay. **A** cerebrospinal fluid (1:3.2); **B** serum (1:100)

Brain MRI showed a few speckled abnormal enhanced signal shadows in the right insular lobe, suggesting inflammatory changes (Fig. 1). Abdominal CT indicated a mass in left retroperitoneal (Fig. 2). Resection of the mass in left retroperitoneal was performed, and the pathological immunohistochemistry was consistent with gangliocytoma (Fig. 3). Cerebrospinal fluid (CSF) analysis showed WBC count 90.0 × 10^6/L with a lymphocytic predominance, normal levels of protein and glucose. The oligoclonal bands both in the CSF and serum were positive. The IgG content in CSF and serum was 135.0 mg/L (10-30 mg/L) and 31.67 g/L (7–16 g/L) respectively. Electroencephalogram (EEG) demonstrated no epileptiform discharges or seizures. Serum and CSF autoimmune encephalitis antibodies, including anti-NMDAR, anti-AMPAR1, anti-AMPAR2, anti-LGI1, anti-CASPR2, anti-GABABR, anti-DPPX, anti-igLON5, and anti-GAD65 were all negative by cell-based assay, with the exception of anti-mGluR5 being positive either in the cerebrospinal fluid (1:3.2) or serum (1:100) (Fig. 4), while tissue-based assay in monkey cerebellum was negative. They were all carried out at Guangzhou V-Medical Laboratory Co., Ltd., China using an autoimmune encephalitis IgG antibody kit manufactured by EUROIMMUN AG, Luebeck, Germany. Laboratory indirect immunofluorescence procedures are as follows: 1. Preparation: The samples and reagent are balanced to room temperature. 2. Incubation: Add samples in the reaction area of sample adding according to the sample adding scheme. Cover the side of the slide covered with biological sheet in the groove of sample adding, and incubate for 30 min at room temperature. 3. Washing: Rinse the slide with a flush of PBS-Tween using a beaker and immerse them immediately afterwards in a cuvette containing PBS-Tween for at least 5 min. 4. Incubation: Apply fluorescein labelled anti-human globulin and incubate at room temperature for 30 min away from light. 5. Washing: same cleaning steps as above. 6. Mounting: Place mounting medium onto a cover glass. Use a polystyrene mounting tray. Put the BIOCHIP slide, with the BIOCHIPs facing downwards, onto the prepared cover glass. 7. Evaluation: Read the fluorescence with the microscope. A package of CSF infectious studies were negative including herpes simplex virus polymerase chain reaction.

The clinical diagnosis was paraneoplastic autoimmune encephalitis. For treatment of AE, the patient received intravenous immunoglobulin (0.4 g/kg/d*5 days) for blocking relevant antibodies, and resection of the gangliocytoma subsequently. Then intravenous methylprednisolone (10 mg/kg/d-5 mg/kg/d-2.5 mg/kg/d for 3 days respectively). Oral prednisone was then initiated, starting at 2 mg/kg/d and the amount was gradually reduced. After 1 month of treatment, the patient's mental symptoms improved significantly and was discharged from hospital.

However, the patient re-hospitalized again on June 28, 2022 due to poor medication compliance. The brain MRI showed no encephalitis lesions. The mGluR5 antibody IgG in serum and cerebrospinal fluid were re-tested and no obvious changes were shown. Abdominal ultrasonography was normal. The IVIg (0.4 g/kg/d*5 days) and glucocorticoids (500 mg/d-400 mg/d-300 mg/d-200 mg/d-100 mg/d for 2 days respectively). Oral prednisone was then initiated, starting at 2 mg/kg/d and the amount was gradually reduced. He was discharged after condition improved significantly. Follow-up revealed the symptoms of patient were relatively stable, with occasional hallucinations mentioned.

## Discussion

The mGluR5 is a pro-metabolic glutamate receptor, located in the postsynaptic terminal of neurons and microglia cells, which is an important mediator of excitatory synaptic transmission. It mainly expresses in the amygdala and hippocampus [3, 4], and plays a role in memory and behavioral learning [5, 6]. The mGluR5 antibodies belong to the antineuronal cell surface antigen antibodies, binding to mGluR5 on the cell surface, resulting in the decrease of mGluR5 cluster density, which is speculated to be the possible pathogenic mechanism. However, the exact mechanism of mGluR5 is still unknown [7, 8].

At present, only few cases of anti-mGluR5 encephalitis described (18 patients in total, only 5 children among them) worldwide, which occurred at different ages, but the median age was 35 years [2, 7, 9–17]. The observed clinical phenotype previously reported showed that most cases were subacute onset, including prodromal symptoms such as low fever, weight loss, headache, respiratory system and digestive system diseases. Limbic system symptoms were mainly manifested, including seizures, cognitive and mental disorders, sleep disorders, language disorders, movement disorders, etc.

In our study, the patient was a 12-year-old boy with subacute onset, with headache, low fever and other prodromal symptoms. The neurological symptoms mainly included mental disorders (hallucinations, mood changes, etc.) and sleep disorders. Among the 18 reported cases, mental symptoms was the main manifestations (90%), but the child had no obvious seizures, cognitive disorders, movement disorders and cranial nerve involvement, which were quite unique compared with previous reported cases. For cerebrospinal fluid of patients of anti-mGluR5 encephalitis, the number of white blood cells was increased, and the specific oligoclonal zones were mostly positive. Electroencephalogram in some patients may have abnormal findings, such as diffuse or localized slow wave, visible epileptic discharge. MRI in some patients may be positive, with limbic system lesions, but thalamus, pontine, cerebellum and fronto-parietal occipital lobe may also be involved. Cerebrospinal fluid examination of this patient showed pleocytosis (> 5 × 10^6^/L) and lymphocytic inflammation. Cerebrospinal fluid oligonclonal zone was positive, and MRI showed a few abnormal enhanced signal shadows in the right insular lobe, which consistent with the manifestations of marginal lobe encephalitis. However, no significant abnormalities in multiple electroencephalograms were found in this patient, which was different as previous reported cases. It suggests that autoimmune encephalitis may not result in EEG background wave slowing and epileptic wave release. After first-line immunotherapy (immunoglobulin combined with glucocorticoid), ganglion cell tumor resection and other treatments, the symptoms of the child were gradually relieved. During follow-up, the neurological symptoms were recurrence, but they were relieved after active treatment again. The recurrence may be related to the low dose or short course of the first methylprednisolone shock therapy. Among the previously reported cases, 2 patients had recurrent neurological symptoms during follow-up, including 1 patient with tumor recurrence. Overall, all reported cases achieved complete or partial remission except for one death, suggesting prognosis of this disease is good. However, close follow-up is necessary in order to pay attention to neurological symptoms and the possibility of tumor recurrence. Clinical information, results from ancillary tests, treatment, and outcome at last follow-up in 6 children are detailed in Table 1.

**Table 1:** Main clinical features, examination, treatment and prognosis in 6 children with anti-mGluR5 encephalitis

Patient sex, age (y)	Prodromal features	Main clinical features	Tumor	CSF analysis	MRI	Treatment	Last follow-up, mo; outcome;
Patient1 M,15	Headache, nausea	Confusion, auditory and visual hallucinations, decreased verbal output, attention deficit, status epilepticus	Hodgkin's lymphoma	114 WBC, OCB	Bilateral (left > right) posterior cortical diffusion restriction	Chemotherapy, RT	72; Complete recovery
Patient2 F,16	Headache	Psychosis, hallucinations, poor sleep, dystonia, generalized seizures	Hodgkin's lymphoma	31 WBC, OCB	Normal	Steroids, chemotherapy, PE	48; Symptoms of Neurological symptom and tumor recurrence
Patient3 F,6	Rash, headache, flulike symptoms	Status epilepticus, dLOC, memory loss, poor sleep with altered sleep–wake cycle, followed by dystonia and oculogyric crisis, psychomotor slowness, ataxia, speech and motor regression, hypoventilation	None	21WBC OCB negative	Bilateral frontal (left > right) and right occipital lobes, cerebellum	Steroids, IVIg, RTX	19; Partial, improved aphasia, cannot walk unassisted
Patient4 M,15	None	Facial paralysis, then developed altered behavior, memory loss, anxiety, irritability, visual hallucinations, insomnia	Hodgkin's lymphoma	45 WBC, OCB	Normal	Steroids, IVIg, chemotherapy	12; Moderate memory problems
Patient5 F,15	loss of appetite	Psychobehavioral abnormalities, seizures, generalized myoclonus	None	Normal	Mild cerebral atrophy	Steroids, IVIg	3; Lethargy
Patient6 M,12 this case	headache, intermittent fever	hallucination and auditory hallucination, mental disorders, sleep disorder	gangliocytoma	90.0 WBC, OCB	encephalitis in limbic	Steroids, IVIg resection of gangliocytoma	3;occasional hallucinations mentioned

*Abbreviations:* dLOC decreased level of consciousness, IgG immunoglobulin G, IVIg IV immunoglobulin, mGluR5 metabotropic glutamate receptor 5, OCB CSF oligoclonal bands, PE plasma exchange, RT radiotherapy, RTX rituximab, WBC white blood cells per mm

In addition, among the 18 cases reported so far, more than half of them were accompanied by Hodgkin's lymphoma, and 1 case was small cell lung cancer. The mechanism by which Hodgkin's lymphoma or other malignancies are associated with autoimmune encephalitis is not well understood. In fact, in the majority cases of autoimmune encephalitis, a clear triggering factor is elusive. There is evidence from cohort studies that age, ethnicity, and HLA type may increase susceptibility [18]. Recently, Guo et. al reported that over half of the Western patients with anti-mGluR5 encephalitis had associated tumors (mainly Hodgkin’s disease), however, only 13% of Chinese patients had associated tumors, and none of them had Hodgkin’s disease at the last follow-up [19].

## Conclusions

Taken together we report the first patient with anti-mGlur5 encephalitis complicated with gangliocytoma. It suggests that in addition to paying attention to the common lymphoma associated with anti-mGlur5 encephalitis, we should also screen the possibility of other tumors for early detection of the cause, active treatment and prevention of recurrence.

## Data Availability

The datasets used and/or analysed during the current study are available from the corresponding author on reasonable request.
